# Role of the clarithromycin immune modulator activity on abdominal microhemodynamics and mortality in severe sepsis

**DOI:** 10.1186/cc11781

**Published:** 2012-11-14

**Authors:** FRE Jesus, CD Snak-Souza, FM Dulcini, EC Del-Massa, LR Gondim, AMA Liberatore, IHJ Koh

**Affiliations:** 1 Federal University of São Paulo, Brazil

## Background

The management of the uncontrolled systemic inflammatory response in the early phase of severe sepsis is still a challenge. Herein we aimed to minimize the acute inflammation phase of sepsis by clarithromycin associated with intravascular volume restoration with an aggressive fluid therapy.

## Methods

Wistar rats were distributed into five groups: S, animals submitted to sepsis (DL_80_; 3 ml *Escherichia coli *10^9 ^CFU/ml, i.v., *n *= 6); SCH, animals induced to sepsis after clarithromycin (14 mg/kg, i.v., given 24 and 0 hours before sepsis) and hyperhydration (Ringer lactate 40 ml/kg, i.v., in 20 minutes after the sepsis induction) (*n *= 6); SC, animals induced to sepsis and treated with clarithromycin (*n *= 6); SH, animals induced to sepsis and treated with hyperhydration (*n *= 6); and Sham, animals induced to sham sepsis (*n *= 4). All invasive procedures were undergone after general anesthesia. The liver, kidney and ileum microcirculation were monitored by Laser Doppler and sidestream darkfield imaging at 2-hour and 26-hour periods. The mortality index was observed for 30 days.

## Results

All animals of the SCH and Sham groups survived while the S, SC and SH groups showed 20% survival. Hyperhydration or clarithromycin in sepsis showed a partial and transient improvement of the microcirculation of the abdominal organs, although the association of hyperhydration with clarithromycin (SCH group) showed better effects. In addition, the beneficial microhemodynamic effect under combined therapy was better evidenced in the liver and intestine as compared with the kidney.

## Conclusion

The association of clarithromycin and hyperhydration showed a beneficial effect in severe sepsis, possibly by modulating inflammatory response and microcirculation damage, respectively, resulting in subsequent survival; nevertheless, their individual effects were not efficient to inhibit severe sepsis mortality and microcirculation dysfunction.

